# Annals of Surgery

**Published:** 1891-02

**Authors:** 


Annals of Surgery. A monthly review of Surgical Science and Practice, edited by L. S. Pilcher, A. M., M. D., of Brooklyn, N. Y., and C. B. Keetley, F. R. C. S., of London, England. Published by J. H. Chambers & Co., 914 Locust Street, St. Louis, Mo. Subscription price, $5.00 per year in advance.

We cannot too highly commend this journal to our readers. It is the only journal now issued which is devoted exclusively to surgery. The articles are from the pens of the most eminent surgeons. Full details of the most wonderful operations are given, frequently with illustrations.

Every physician, who is in active practice, should take it, as it most certainly keeps him informed of all the latest developments of surgical art.

It is elegantly printed, with leaded lines on fine paper, and is well worth the subscription price.

Some of our subscribers have complained that there is not enough attention given to surgery in the pages of The Homœopathic Physician. To all such we cordially recommend the *Annals of Surgery* as leaving nothing to be desired on that subject. W. M. J.

